# Reappraising the Value of HIV-1 Vaccine Correlates of Protection Analyses

**DOI:** 10.1128/jvi.00034-22

**Published:** 2022-04-06

**Authors:** P. J. Klasse, John P. Moore

**Affiliations:** a Department of Microbiology and Immunology, Weill Cornell Medicine, New York, New York, USA; Emory University

**Keywords:** COVID-19, correlates of protection, HIV-1, SARS-CoV-2, SIV, clinical trials, neutralizing antibodies, nonhuman primates, nonneutralizing antibodies, systems biology

## Abstract

With the much-debated exception of the modestly reduced acquisition reported for the RV144 efficacy trial, HIV-1 vaccines have not protected humans against infection, and a vaccine of similar design to that tested in RV144 was not protective in a later trial, HVTN 702. Similar vaccine regimens have also not consistently protected nonhuman primates (NHPs) against viral acquisition. Conversely, experimental vaccines of different designs have protected macaques from viral challenges but then failed to protect humans, while many other HIV-1 vaccine candidates have not protected NHPs. While efficacy varies more in NHPs than humans, vaccines have failed to protect in the most stringent NHP model. Intense investigations have aimed to identify correlates of protection (CoPs), even in the absence of net protection. Unvaccinated animals and humans vary vastly in their susceptibility to infection and in their innate and adaptive responses to the vaccines; hence, merely statistical associations with factors that do not protect are easily found. Systems biological analyses, including artificial intelligence, have identified numerous candidate CoPs but with no clear consistency within or between species. Proposed CoPs sometimes have only tenuous mechanistic connections to immune protection. In contrast, neutralizing antibodies (NAbs) are a central mechanistic CoP for vaccines that succeed against other viruses, including SARS-CoV-2. No HIV-1 vaccine candidate has yet elicited potent and broadly active NAbs in NHPs or humans, but narrow-specificity NAbs against the HIV-1 isolate corresponding to the immunogen do protect against infection by the autologous virus. Here, we analyze why so many HIV-1 vaccines have failed, summarize the outcomes of vaccination in NHPs and humans, and discuss the value and pitfalls of hunting for CoPs other than NAbs. We contrast the failure to find a consistent CoP for HIV-1 vaccines with the identification of NAbs as the principal CoP for SARS-CoV-2.

## INTRODUCTION

Over the past 15 years, multiple human immunodeficiency virus type 1 (HIV-1) vaccine trials have failed to yield evidence for significant efficacy (Table 1). The outcome of one, the RV144 trial, was statistically marginal protection against infection, but vaccination status had no impact on viral load or disease progression in the follow-on RV152 study (1, 2). Evidence of protection against infection was not found in a subsequent trial, HVTN 702, of a conceptually similar vaccine design (3). The RV144/RV152 and HVTN 702 trials involved priming with poxvirus vectors expressing HIV-1 Gag, Pol, and part of the HIV-1 envelope glycoprotein (Env), to which was added boosting with two different gp120 proteins, the outer Env subunit, in the later immunizations (2). Similarly, negative results have emerged from human trials based on DNA or adenovirus vectors, most recently in the HVTN 705 study in which priming with HIV-1 Gag, Pol, and Env, expressed from an adenovirus vector (Ad26), was followed by a boost with two early-generation, nonnative gp140 Env proteins combined with the vector in the last immunizations (Table 1) (4).

**TABLE 1:** Human HIV-1 clinical efficacy (2B and 3) trials that included Env immunizations given as protein or expressed from vector or both

Study (reference)	Immunogens^*a*^	Vaccine efficacy^*b*^	Proposed CoP^*c*^
VAX004 (50, 116)	AIDSVAX, B/B gp120 × 7, alum adjuvant	NS	
VAX003 (117)	AIDSVAX, B/E gp120 × 7, alum adjuvant	NS	
RV144-RV152 (2, 51, 55, 59)	Prime: ALVAC-HIV (vCP1521) × 4; boost: AIDSVAX gp120 B/E (MN/A244) × 2,^*d*^ in alum adjuvant	31% at 3.5 yr by MITT analysis	High V1V2 Ab reactivity in plasma, low Env-specific IgA in plasma
HVTN 505 (62)	Prime: DNA *gag*, *pol*, *nef*, *env* A/B/C × 3; boost: rAd5 *gag-pol* B, *env* A/B/C × 1	NS	
HVTN 702 (3)	Prime: ALVAC-HIV (vCP2438) × 6; boost: bivalent gp120 C (TV1/1086) × 4,^*d*^ in MF59 adjuvant	NS	
HVTN 705 /HPX2008 (4, 41)	Prime: Ad26 mosaic HIV (*gag*, *pol*, *env*) × 4; boost: gp140 C × 2, in alum adjuvant	NS	

a Single capital letters with or without slashes denote clade of viral gene or protein, e.g., C, B/E, A/B/C.

b NS, nonsignificant VE. MITT, modified intention to treat; protection was not significant in the other protocols with intention to treat and per protocol.

c No tier-2-neutralizing responses, i.e., no bNAb responses were detected.

d Protein boosts were given simultaneously with the last two or four vector immunizations.

Some of the above-described trials were based on the premise that modest CD8^+^ T-cell responses, in combination with nonneutralizing antibodies (non-NAbs), would be sufficient for protection against HIV-1 acquisition or at least to reduce viral loads. There has never been a consensus behind this approach to an HIV-1 vaccine (5); it was questioned throughout the prolonged period of multiple efficacy trials (6–12). In the interval between the RV144 and HVTN 702 trials and during the latter, the evaluation of similar vaccines in nonhuman primates (NHPs) yielded contradictory, but mostly negative, outcomes (13–16). Despite the slender and inconsistent experimental evidence that a vaccine inducing some T-cell responses plus non-NAbs would work, the federal government and charitable foundations invested huge resources in immunogen design, production, and testing over a multidecade period, generally more so than did the companies that developed the vaccine candidates. It is time for a serious evaluation of where HIV-1 vaccine research currently stands, so as to guide where it should now head.

Protection against viral acquisition usually entails the induction of neutralizing antibodies (NAbs) that are active against the virus strains to which the vaccinated person is exposed (6, 7, 17, 18). We have seen that principle confirmed and extended by the successful coronavirus disease 2019 (COVID-19) vaccines, for which the principal correlate of protection (CoP) is serum NAbs (19, 20). There are differences, of course: HIV-1 is not a respiratory virus, and it is transmitted via the rectal, penile, vaginal, or blood-borne routes rather than by inhaling. Furthermore, NAbs apparently have a greater role in suppressing viral loads in infection with SARS-CoV-2 than with HIV-1 (21–23). Nonetheless, the inability of the multiple HIV-1 vaccines to induce broadly active NAbs is arguably a fundamental limitation on their immunogenicity. Strong and lasting CD8^+^ T-cell responses may provide alternative or complementary protection (24, 25), but here we will focus on antibodies as potential CoPs. We therefore consider only immunization regimens that include Env, delivered either as protein or expressed from vectors. Env is the only antigen anchored in viral and cell surface membranes and is the sole target for NAbs. Thus, we will not further analyze efficacy trials that did not include an Env component but relied only on T-cell responses. Although those non-Env vaccines elicited some cytotoxic T-cell responses to conserved epitopes, they were not protective against HIV-1; indeed, infection rates were higher in some vaccine groups than placebo (26–28). We will also not discuss CoPs for attenuated simian immunodeficiency virus (SIV) vaccines. Those vaccines are no longer being considered for human trials and are a complex topic that deserves its own review.

The human efficacy trials have nearly consistently failed (Table 1). How can that be reconciled with the many inconsistent outcomes in macaque experiments? In the most stringent macaque model protection has also been elusive (Table 2). The conditions of viral challenge in that model may best mimic the formidable demands on immune responses to protect against the transmission of HIV-1 to humans. Here, we analyze the conditions that render searches for CoPs meaningful; we discuss the implications of hypotheses about the causes of protection; we scrutinize systems biology screens for factors statistically associated with reduced infection risk in the absence of overall protection; we analyze the contradictions among these factors and their mechanistic plausibility or lack of it; and we suggest explanations for these findings and why they may not by themselves fruitfully inform vaccine development.

**TABLE 2:** Proposed CoPs in macaque SIV or SHIV challenge models

Reference	Immunogen(s)^*a*^	Challenge virus, dose, route^*a*^	VE^*b*^ (%)	Proposed CoP	NAb titer against challenge virus
Roederer et al. (43)	SIVmac239 Prime: DNA x 3 Boost: Ad5 x 1 IM	SIVsmE660 Dose: 30% infection per exposure in controls 12 iterations IR	69%	NAb titers	40-98% neutralization of variants; ID_50_ (half-maximal inhibitory dilution) against SIVsmE660 sensitive clone CP3C: 10^3^ - 10^6^
Bradley et al. (13)	HIV-1 B/E Prime: ALVAC x 2 Boost: ALVAC + Env B/E or B/E/E/E/E protein x 4 IM	SHIV-1157(QNE)Y173H Dose: low 8 iterations IR	B/E =11% B/E/E/E/E = 56% after 8th challenge, NS	ADCC ^*c*^ MIP-1β	ND ^*d*^
Ackerman et al. (36)	HIV-1 B/E Prime: ALVAC x 2 Boost: ALVAC + Env B/E or B/E/E/E/E protein x 4 IM	SHIV-1157(QNE)Y173H Dose: low 8 iterations IR	B/E B/E/E/E/E Pool 35% after 8th challenge, NS	IgG ADCP ^*c*^	ND (as tested in (13))
Ackerman et al. (36)	SIVmac239 Prime: DNA x 3 Boost: Ad5 x 1 IM	SIVsmE660 Dose: 30% infection per exposure in controls 12 iterations IR	69%	IgG ADCP	ND (as tested in (45))
Ackerman et al. (36)	SIVmac239 Prime: DNA x 3 Boost: Ad5 x 1 Aerosol	SIVsmE660 Dose: 30% infection per exposure in controls 12 iterations IR	70%	IgA ADNP ^*c*^	NA^*d*^
Barouch et al. (64)	SIVsmE543 Prime: MVA *gag, pol* and *env* Boost: Ad26 (or converse order)	SIVmac251 Dose: 930 TCID50 6 iterations IR	80%	V2, Env binding Tier-1 NAbs	<50% extent of neutralization
Barouch et al. (168)	Mosaic HIV-1 g*ag, pol, env* Prime: Ad26/35 x 1 Boost: MVA x 1 or Prime: Ad26/35 x 1 Boost: Ad26/35 x 1 IM	SHIVSF162P3 Dose: 1/100 dilution of stock 6 iterations IR	∼90%	Env binding SF162 NAbs ADCP ADCD ^*c*^ trend	70-110
Barouch et al. (40)	Prime: Ad26 SIVsmE543 *env, gag, pol* Boost: SIVmac32H gp140 IM	SIVmac251 SHIVSF162P3 Dose: 500 TCID50 6 iterations IR	90%	Env binding ADCP	∼30% extent neutralization against SIVmac- 251.30
Barouch et al. (4)	Prime: Ad26 Mosaic HIV-1 g*ag, pol, env* x 2 Boost: Clade C gp140 IM	SHIVSF162P3 Dose: 500 TCID50 6 iterations IR	94%	Clade C gp140 binding, ELISPOT	NA
Fouts et al. (171)	HIV-1 Ba-L Prime: gp120-CD4 chimera or gp120 x 2 IM or DNA *gp120-CD4 gag, pol* x 3 Boost: gp120-CD4 chimera or gp120 x 2 IM	SIVmac251 or SHIV162P3 Dose: 50 (or 50, 100, and 200) TCID50 14 iterations IR	∼70%	ADCC when T-cell responses were low	ND
Bogers et al. (115)	HIV-1 89.6, SF162 Prime: Ad5hr *env* x 2 IN or IT Boost: SF162 gp140 protein or SF162 gp140ΔV2 alphavirus x 2 IM	SHIV-SF162p4 Dose: 1800 TCID50 IR	50-75% based on final outcome	NAb titers on day of challenge ADCC	Protected >80 Unprotected <70
Miller- Novak et al., Tuero et al. (79, 85)	SIV various Prime: Replicating Ad SIVsmH4 *env/rev*, SIV239 *gag* and SIV239nefΔ1–13 x 2 IN and OR then IT Boost: SIVmac239 monomeric gp120 or oligomeric gp140 x 2 IM	SIVmac251 Dose: 120 TCID50 9 iterations IR	NS overall delay in infection (current controls); significant delay for females only	Rectal IgA to Env overall Rectal Env-specific memory B and plasma cells in females Virion but not cell lysis by ADCML in males	ND: Only detected against a sensitive version of challenge virus; no sex difference
Xiao et al. (86)	SIV various Prime: Replicating Ad5 SIVsmH4 (*env*) SIVmac239 (*gag*) x 2 s.l. or IN then IT or IV or IR Boost: SIVmac251gp120 x 2 IM	SIVmac251 Dose: 130 TCID50 9 iterations IR	NS delay; one IR-immunized animal uninfected	Antibody avidity (chaotrope assay) ADCC Rectal sIgA	ND
Sui et al. (92)	Prime: MVA SIVmac239 *gag, pol, env, tat,* and *nef* x 2 OR Boost: HIV-1 gp120-CD4 chimera on NP x 2 OR (most successful of several regimens)	SHIV SF162.P4 Dose: high or low 8 iterations IR	44%	Gut microbiome alteration; trained innate immunity; possibly virus-specific T cells	ND, no Env binding
Letvin et al. (65)	SIVmac239 Prime: DNA *env* and *gag-pol* x 3 IM Boost: Ad5 *env* and *gag-pol* x 1 IM	SIVmac251 or SIVsmE660 Dose: 1 AID_50_ 12 iterations IR	∼50% against SIVsm660; none against SIVmac251	Neutralization (%) at 1/50 serum dilution CD4L+ T-cells	Extent of neutralization at 1/50 serum dilution (∼90% in uninfected, 20% in infected Mamu-A*- animals)
Helmold Hait et al. and Hunegnaw et al. (90, 91)	SIV various Prime: Replicating Ad5 SIVsmH4 *env/rev* SIVmac239 *gag x 2* IN-or. then IT Boost: SIV M766 and later CGTV gp120 x 2 IM	SIVmac251 800 TCID_50_ 12 iterations IV	NS delay	ADCC but not ADCP FcγRIII Expression in cervico-vaginal macropahges	NA
Musich et al. (89)	SIV various Prime: Ad5hr SIVmac239 *gag* SIVM766 *gp120-TM* x 2 OR-IN then IT Boost: ALVAC-SIVM766 *env/gag/pro* + SIVM766 & CG7V gp120 x 2 IM or DNA SIVM766 *env* SIVmac239 *gag* macaque *IL-12* + SIVM766 & CG7V gp120 x 2 IM	SIVmac251 120 TCID_50_ 15 iterations IR	NS delay	Env-specific rectal IgA/total rectal IgA in all vaccinated animals (r = 0.35) ADNP in male vaccinees Changes in gut microbiome, greatest in females	NA: Only tested against neutralization-sensitive viruses
Vaccari et al. (15)	SIVmac251 & SIVsmE660 Prime: ALVAC SIVmac251 *gag-pro* and *gp120TM* x 2 IM Boost: ALVAC SIVmac251 *gag-pro* and *gp120TM* + SIVmac251(M766) & SIVsmE660 (CG7V) gp120 in Alum or MF59 adjuvant x 2 IM	SIVmac251 low dose 10 iterations IR	Alum group: 44%; MF59 group: NS delay	Alum group: Env-stimulated IL-17 secretion from innate lymphoid cells, expression of 12 genes, 10 in the RAS pathway, rectal V2-specific IgG; MF59 group: *rectal IgG to V2: higher risk*	NA: Only tested against neutralization-sensitive variant of SIVmac251
Vaccari et al. (172)	SIVmac251 & SIVsmE660 Prime: DNA x 2 or Ad26 x 1 IM Boost: ALVAC SIVmac251 *gag-pro* and *gp120TM* + gp120-SIVmac251(M766) and gp120 SIVsmE660(CG7V) x 2 IM	SIVmac251 Dose: low 10 iterations IR	DNA group: 52%; Ad26 group: NS delay	Hypoxia and inflammasome in CD14+ monocytes DNA group: Rectal IgG to cyclic V2 corr. but not higher levels than in Ad26 group; *V1V2-binding IgG in serum and rectal secretions higher for Ad26 than DNA prime*	NA: Only tested against neutralization-sensitive variant of SIVmac251
Schifanella et al. (16)	HIV-1 B/C Prime: ALVAC SIVmac *gag-pol* + ALVAC-HIV *gag-pro-env* x 4 or 5 IM Boost: gp120 (Clade C TV1+1086) x 2 or 4 Low-dose Alum, High-dose Alum or MF59 adjuvant IM	SHIV-C (1157ipd3N4), neutralization-sensitive 12 or 17 iterations IV	Low-dose Alum: NS; high-dose Alum: NS MF59: 64%	Alum low dose: IgA to V2 Increased risk Alum high dose: IgG to V2 Decreased risk MF59: NAbs against SHIV-C (Tier-1)	∼20-100
Kwa et al. (173)	SIVmac239 Prime: DNA *gag-pol-env-tat-rev* +/- CD40L IM Boost: MVA *gag-pol-env-tat-rev* +/- CD40L IM	SIVmac251 low dose 8 iterations IR	+ CD40L group: 50%; - CD40L group NS delay	Fewer linear epitopes recognized in V1 and gp41; stronger V2 response in +CD40L than -CD40L group	ND
Pegu et al. (14)	SIV mac251K6W Prime: ALVAC *gag-pol* (vCP172) *env* (vCP1420) x 4 IM Boost: gp120 x 2 IM	SIVmac251 120 TCID_50_ 6 iterations IR	NS delay	High avidity of gp120-specific IgG; V1V2-specific Ab (not powered for CoP analysis)	NA: Only tested against neutralization-sensitive variant of SIVmac251
Strbo et al. (174)	SIVmac251 Irradiated HEK293 cells transfected with gp96 SIVmac251 *rev-tat-nef, gag, and env* x 3 + SIV rgp120 x 2 IP	SIVmac251 120 TCID_50_ 7 iterations IR	73%	SIVmac251-specifc Abs and CTL	ND
Gonzales-Nieto et al. (47)	SIVmac239 and 316 Prime: DNA near-full-genome (E767 stop) x 4 IM Boost: 5 RRVs with SIVmac inserts) x 1 or x 2 IVE	SIVmac239 (clonal) 200 TCID_50_ 6 iterations IR	78%	None identified	Weak or ND
Martins et al. (48)	SIVmac239 and 316 Prime: 5 RRVs with SIVmac inserts) x 2 IVE then IVE-OR Boost: DNA near-full-genome (E767 stop) x 4 IM	SIVmac239 (clonal) 0.3-0.5 AID_50_ 6 iterations IVE	79%	None identified	ND
Martins et al. (49)	SIVmac239 entire proteome: DNA x 3 (IM-EP); MVA (IVE); VSV (IVE); Ad5 (IM); RRV (IVE); DNA x 4; (IM-EP): 11 immunizations over 74 weeks	SIVmac239 (clonal) 200 TCID_50_ 6 iterations IR	NS	NA	ND except SIVmac239 NAb ID_50_ ∼30 in one monkey, infected upon first challenge
Arunachalam et al. (149)	HIV-1 BG505 SOSIP.664 trimers x 4; SC *+/-* HVV-*gag* x 3 IVE	SHIV- BG505.332N.375Y 10 iterations IV	After 10 challenges: 53% - HVV-*gag* 67%; +HVV-*gag*	NAbs in - HVV-*gag* group; NAbs and Gag-specific CD8+ in + HVV- *gag* group	Protective: >300 - HVV- *gag*; <300 + HVV- *gag*
Pauthner et al. (150)	HIV-1 BG505 SOSIP trimers x 3 SC	1.4 x 10^7^virions BG505.332N.375Y 12 iterations IR	100% in highest NAb group	Autologous NAb ID50 >500 against pseudovirus	High and low autologous NAb-titer animals were selected
Bomsel et al. (175)	HIV-1 HxB2 gp41 peptides coupled to virosomes IM x 4 or IM x 2 then IN x 2	SHIV-SF162P3 20-30 TCID_50_ 13 iterations IV	After 13 challenges: IM group: 50% IM+IN group: 100%	Transcytosis-blocking mucosal IgA ADCC by mucosal IgG	No neutralization by serum; cervico-vaginal fluid neutralized HIV-1 JR-CSF
Zhang et al. (167)	mRNA VLP WITO N276 KO x 1 Different clades mRNA VLP or Env trimer protein x 9 IM	SHIV AD8 10 TCID_50_ 13 iterations IR	79%	bNAbs to CD4-binding site	10-100

a IM = intramuscular; IN = intranasal; IT = intratracheal; SL = sublingual; IP = intraperitoneal; IV = intravaginal; IVE = intravenous; OR = oral; EP = electroporation; SC = subcutaneous; NP = nanoparticle; ALVAC = Canarypox-viral vector; MVA = modified vaccinia Ankara; VV = vaccinia virus; VSV = vesicular stomatitis virus; Ad5 = Adenovirus 5; RRV = rhesus rhadinovirus; HVV = heterologous viral vectors: VV, VSV and Ad5. TCID50 = tissue culture infectious dose; AID50 = animal infectious dose; VLP = virus-like particle; KO = knock-out.

b Some studies also evaluated effects on the viral loads (VL). Here, the focus is on protection against acquisition and on studies that have analyzed associations with the number of challenges needed for infection, even in the absence of net protection against acquisition. When calculated from Kaplan-Meier plots for decreasing uninfected status with increasing number of challenges, the term (incidence of infection among vaccinees)/(incidence of infection among controls) is the hazard ratio derived from a Cox regression model; the resulting VE is the efficacy per challenge. For some studies (13, 38, 115), which lack these analyses, VE was instead calculated on the basis of the final outcome as indicated; some research groups calculate both kinds of VE; i.e., per challenge and after a certain number of challenges (42, 170). NS = non-significant.

c ADCC = antibody-dependent cellular cytotoxicity; ADCP = antibody-dependent cellular phagocytosis mediated by monocytes; ADNP = antibody-dependent neutrophil-mediated phagocytosis; ADCVI = antibody-dependent cell-mediated viral inhibition; ADCML = antibody-dependent complement-mediated lysis (of cells or virions); ADCD = antibody-dependent complement deposition. ASC = antibody-secreting cells; OD = optical density

d ND = Not detectable; NA = not analyzed.

## WHAT ARE CORRELATES OF RISK AND PROTECTION?

**FIG 1:**
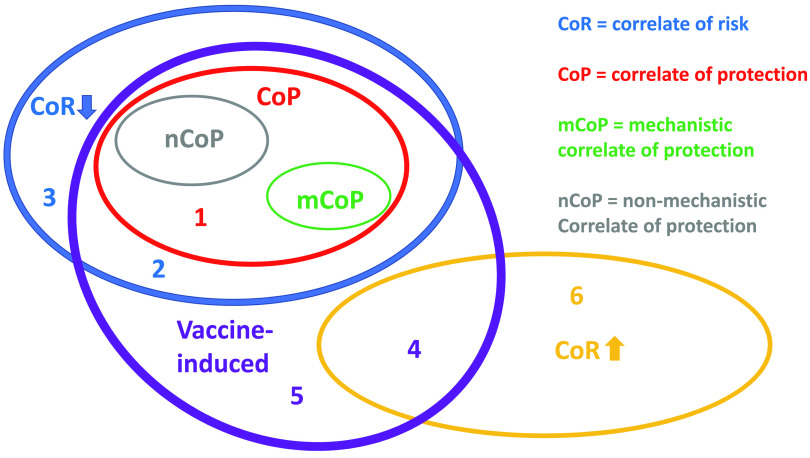
Nomenclature of correlates of risk and protection. The diagrams show two nonoverlapping correlates of risk (CoRs): correlates of increased risk (yellow, upward arrow) and decreased risk (blue, downward arrow). Correlates of protection (CoPs) are a subset of correlates of decreased risk. CoPs have the added inclusion criteria of being vaccine induced and associated with net protection in the vaccine group or at least in a well-defined subgroup. CoPs can be mechanistic (mCoPs) or nonmechanistic (nCoPs). Each digit (colored) signifies the entire field or intersection in which it is placed that can be reached without crossing any elliptical line. Thus, the field marked 1 lacks any elements because all CoPs are either mCoPs or nCoPs. As the latter are also mutually exclusive, their ellipses do not overlap (30). A complication is that an mCoP can be a combination of factors, as discussed elsewhere (29). Components in such a combination that are completely inert on their own (coalism) could be designated nCoPs if they correlate with protection. If the components are additive or synergistic, they could conveniently be classified as weak mCoPs on their own and as a strong mCoP in combination (29). Field 2 can be populated. Thus, vaccine-induced factors can be correlates of reduced risk in the absence of net protection, although that absence disqualifies them from being CoPs. Field 3 contains factors that correlate with reduced net or subgroup risk but are not affected by the vaccine. Field 4 contains factors induced by the vaccine that inadvertently cause or are just statistically associated with an increased risk of infection. Field 5 contains vaccine-induced factors that do not affect the risk of infection. Field 6 contains factors that are unaffected by the vaccine but correlate with increased risk of infection, e.g., receptor expression, target cell densities, and coinfections. If, for example, a borderline VE is reclassified as insignificant because of new evidence, such as lack of reproducibility, then the previous putative CoPs would, by definition, move out of their respective nCoP and mCoP ellipses into field 2. Tightened criteria for what is vaccine induced yield other examples of field swapping. Thus, if the only difference between the vaccine and the placebo group were the HIV-1 genetic sequence or protein, “vaccine-induced” could be defined as induced by those HIV-1 components. If, upon stringent new testing, previously vaccine-attributed CoRs turn out to be induced by the control vector or adjuvant in the placebo immunizations, that would move elements out of the nCoP and mCoP fields into field 3. Similarly, the risk-lowering factors, not associated with net protection, in field 2 would move into field 3, i.e., outside the vaccine-induced field. Any CoRs for elevated risk in field 4 would move into field 6.

Multiple terms for variables associated with outcomes of vaccination have been used inconsistently, but the redefinition of correlates of risk (CoR) and of protection (CoP) has clarified the nomenclature (29, 30) (Fig. 1). A CoR is associated with reduced or increased risk and does not have to be vaccine - induced; CoPs are a subset of correlates of reduced risk; a CoP must be altered by vaccination, and at least in a subgroup it must be associated with significant vaccine efficacy (VE), which is defined as

VE = 1 − [(infection rate among vaccinees)/(infection rate among controls)]×100%

**FIG 2:**
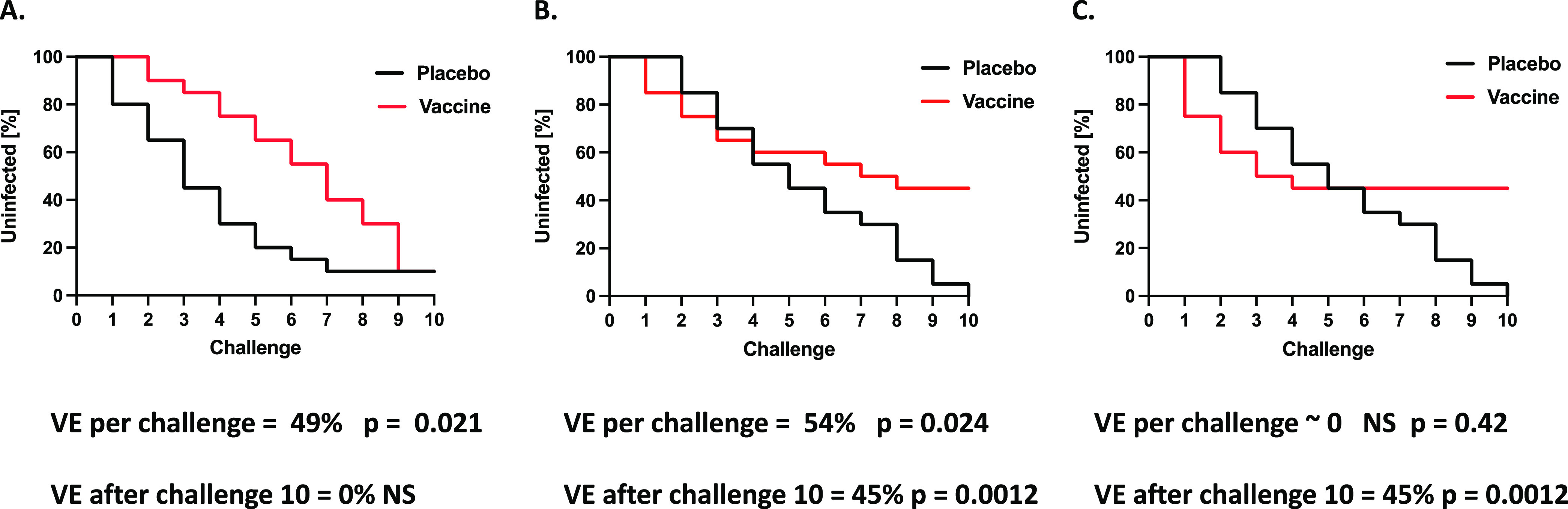
Different bases for calculation of vaccine efficacy. In experimental animal models of HIV-1 infection with iterated viral challenges, the diminishing fraction of uninfected animals can be presented in Kaplan-Meier plots. The remaining fraction of uninfected subjects over time can be similarly plotted in clinical trials. A log rank test can determine whether protection was significant in the vaccine group compared with the placebo controls (*P* values). Vaccine efficacy (VE) can be calculated for the entire curve based on log-rank hazard ratios. Alternatively, VE can be based on the fractions of uninfected subjects at a particular time, e.g., at the last challenge or early and late after vaccination (2, 51, 168). The significance of the difference between the placebo and vaccine recipients can then be determined by Fisher’s exact test. The Kaplan-Maier plots for simulated data in panels A, B, and C illustrate how the two methods can yield widely divergent VE values. In panel A, the protection per challenge is significant but VE = 0 at the end of the experiment (challenge-10). Similarly to this simulated example, in one macaque vaccine experiment with Ad26-SIV Env/Gag/Pol prime and SIV Env gp140 boost, the VE for the pool of vaccinated macaques was substantial and significant on a per-challenge basis (57%, log rank hazard ratios; *P* = 0.02), whereas there was no significant efficacy after 6 challenges (13%, *P* = 1.0, Fisher’s exact test; note that these are our calculations for the pool of vaccine recipients, which was not compared with control animals in the original study [31]). In panel B, VE per challenge is significant and somewhat greater than that in panel A, but the major difference is that it remains substantial after challenge-10. In panel C, VE per challenge is nonsignificant but VE after challenge-10 is substantial and equal to that in panel B.

VE calculations can be based on experimental infection rates estimated at a fixed time (e.g., after the last challenge in a planned series) or, alternatively, on a per-challenge basis (Fig. 2). Thus, after vaccination of macaques in one study, VE on a per-challenge basis was substantial, whereas it was negligible after six challenges (31); a similar outcome with simulated data is illustrated in Fig. 2.

A CoR can be hypothesized to be a CoP, but a CoR is not a CoP if it merely reflects the extent of exposure to a pathogen or the susceptibility of the trial participant to infection. Mechanistic CoPs (mCoPs) measure the protective immune variable itself; a nonmechanistic CoP (nCoP) correlates with the mCoP but does not itself protect. A factor instrumental in protection also need not be sufficient for that protection to occur, a distinction worth emphasizing. For further discussion of CoPs as links in causal chains, the mutual exclusivity of mCoPs and nCoPs, and the statistics of CoR and CoP analysis for combined factors, see references 29, 30, and 32–35.

The various HIV-1 vaccine efficacy trials were preceded, accompanied, or followed by multiple experiments in NHP models with SIV or SHIV challenge viruses (Tables 1 and 2). Some NHP studies of poxvirus-based vaccines yielded evidence for a reduction in viral load when breakthrough infections occurred but not for protection against virus acquisition (36, 37). It should not have been expected that a vaccine would protect humans from infection, if the best it could achieve in an animal model was a modest reduction of viremia (38, 39). Although borderline protection against acquisition was reported in the RV144 efficacy trial, the viral loads in infected vaccine and placebo recipients were indistinguishable, in contrast to the results in the macaque experiments (Table 1) (1, 2). As noted, there was no vaccine-mediated effect on disease course in the RV152 extension phase of the RV144 trial (1). The reduced viral loads in macaques have not, therefore, been reproduced in humans. Conversely, the marginally reduced acquisition in the RV144 human trial (2) was replicated in one macaque experiment of similar design (15) but not in others (13, 14, 16).

Adenovirus vector vaccines, carrying HIV-1 or SIV genes, including *env* with Env protein boosting, have provided significant protection from acquisition in some macaque experiments (4, 31, 40). The protection was largely attributed to non-NAbs, with support from a macaque challenge study of passive immunization with IgG purified from vaccinated animals that had immune signatures associated with protection (31). However, when humans were immunized with similar adenovirus-based vaccines before Env protein boosting, they were not protected from infection (HVTN 705/HPX2008) (Table 1) (4, 41).

Taken together, the outcomes of NHP and human experiments are sometimes contradictory, sometimes consistent. When they do diverge, several questions arise. Are the demands on the protective vaccines different in the two species? Do vaccine-induced immune responses qualitatively or quantitatively differ between the species? Does virus challenge (rectal or vaginal) of macaques adequately simulate human exposure to HIV-1? Hence, the infections of humans and macaques may differ in many respects that could explain the discrepant outcomes of vaccine experiments in NHPs and efficacy trials in humans. Since the interspecies comparisons are complex and the results subject to stochastic influences, some causes of the different outcomes may never be pinpointed. We note, however, that the protection afforded in NHP experiments strongly depends on the challenge dose (42). Protection may also be influenced by the cell type and method (infection or transfection) used to produce the challenge virus, by selection for truncation of the cytoplasmic tail of Env, and by the heterogeneous neutralization sensitivity of the polyclonal challenge virus (43–46). Most important of all may be the choice of the challenge virus, particularly whether it is homogeneously neutralization resistant and heterologous. In the highly stringent macaque model of infection by clonal neutralization-resistant SIVmac239, even autologous protection was elusive after multiple immunizations with different replicating vectors carrying near-full-length SIV genome inserts (47–49). In conclusion, when the bar is raised in the NHP model by the use of a clonal, neutralization-resistant, heterologous challenge virus, there is rarely, if ever, a major discrepancy between human trials and NHP experiments. In both settings, the general outcome is little or no protection. Furthermore, the negative outcomes in NHP experiments occurred even in the absence of complications such as the immense natural variability in Env and exposure to variable, but sometimes high, doses in human infection (47–49). It is therefore arguable that even the most stringent NHP models sometimes pose lower demands on protection than what is required to prevent human transmission of HIV-1. Against this background, it is now urgent to assess which NHP vaccine experiments best predict human protection against HIV-1.

## CORRELATES OF PROTECTION IN NHP AND HUMAN STUDIES

A consistent focus in NHP immunization and challenge experiments has been to search for CoPs and CoRs; data from human vaccine trials have been similarly analyzed. The rationale is to identify protective or at least beneficial immune responses for elicitation by the next generations of vaccines. Per definition, the determination of a CoP requires there be some protection, at least in a subgroup. If there is neither protection nor net enhancement of infection, it is possible to postulate the occurrence of finely counterbalancing infection-enhancing and infection-reducing effects (i.e., CoRs). However, such effects may not be vaccine specific and, hence, should be investigated among both vaccinees and controls (29, 30, 50). Might an experiment that is insufficiently powered to detect overall protection still give hints about CoPs? That possibility could, in practice, be dismissed: the lack of statistical power would also compromise any identification of a major CoP that acts unopposed. We return to this theme in more detail below.

It is risky to identify CoPs when protection is weak, a difficulty underpinning the inconsistency among the multiple CoP candidates that have emerged to date. Consistency in outcome is lacking both among studies within each species and between macaque and human studies of similar vaccines (Tables 1 and 2). For example, an analysis of RV144 trial data identified non-NAbs to the gp120-V1V2 region as a CoP (on the assumption that the marginal protection was real; if it was not, it would only have been a CoR; Fig. 1), while serum IgA against gp120 was a correlate of increased risk (51). Later analyses suggested that V3-specific antibodies also were protective (52). Vaccine regimens based on those CoP and CoR analyses have not fared well in NHP experiments or follow-on human trials (Tables 1 and 2). Indeed, vaccine designs comparable to that of the RV144 trial completely failed to protect against virus acquisition in humans and have given variable results in macaques (Tables 1 and 2). The conflicting outcomes reinforce long-standing questions about the value of any CoP that is only weakly correlated with low-level protection.

The robustness of the low-level protection in RV144 has been debated, and so have the linked analyses of CoPs and viral mutant selection (i.e., sieving) effects (2, 51, 53–58). We are not relitigating these controversies here, but we emphasize that redesigning and testing vaccines are highly complex and extremely expensive endeavors; the production of immunogens for human use is particularly costly. Given how strongly the RV144 trial influenced the course of HIV-1 vaccine research for so long, independent historians of science may one day question whether the right decisions were made. Substantial effort focused on inducing non-NAbs to a short stretch of one highly variable region, V2, of gp120. The rationales were that V1V2-specific antibodies were identified as the strongest CoP in RV144 and that sieve analyses of transmitted virus were interpreted as vaccine-induced immune pressure on subregions of V2 (51, 55, 59). However, the wider knowledge of Env and its immunogenicity suggests that such antibodies do not neutralize because they do not bind to native Env trimers, cross-react poorly, allow easy escape, and are elicited by gp120 along with antibodies of multiple other specificities (6, 9, 10, 12, 17, 60, 61). It has remained unexplained why a subset of non-NAbs to V2 would distinguish themselves from the others and how they would uniquely confer protection. To avoid future disappointments, if any CoP candidates are identified in more recent efficacy trials they should be thoroughly validated as effective mCoPs experimentally before forming the basis of new vaccine strategies (4, 41, 59, 62, 63) (Table 1).

## THE SEARCH FOR NON-NAb CoPs IN NHP EXPERIMENTS

Three of the numerous NHP challenge studies, one overlapping the other two, serve to illustrate both the complexity of the search for CoPs and the potential for catching redundant or indirectly acting factors (i.e., nCoPs) through extended follow-up analyses (13, 36, 43). The literature in this area is so extensive that we cannot comprehensively dissect it. Suffice it to say that no consistent CoPs have emerged that we are aware of.

Roederer et al. used a SIVmac239 sequence-based DNA prime, recombinant Ad5-*env* boost to immunize macaques intramuscularly and recorded 69% efficacy against iterated challenges with the heterologous SIVsmE660 (43). NAbs became detectable against some variants in the quasispecies of the challenge virus. The breakthrough virus consistently had a neutralization resistance signature in Env, *viz.*, one or both of gp120 residues 45A and 47K. The authors’ straightforward interpretation was that the primary mechanism of protection was NAb-mediated reduction of the effective infectious dose, i.e., virus neutralization *in vivo*. They also suggested that these findings explained the weak or absent correlations between neutralization and protection in earlier experiments in which SIVmac251 was the challenge virus, since that virus invariably contains the 45A/47K signature associated with breakthrough infections (64, 65).

Ackerman et al. then sought potentially complementary CoPs by extended analyses of serum samples from the macaques that had been immunized intramuscularly with SIVmac239 DNA/Ad5-*env*. They also added a study group in which the animals received the same vaccine intranasally as an aerosol and were protected to a similar extent, 70% (36). To define humoral CoRs they used a Cox proportional hazards model and, after validation, identified four features as CoR candidates for the two groups pooled. Correlates of reduced risk were the capacities of V1 and C1 peptide-specific antibodies to ligate rhesus FcγR2A.4 and of SIVmac239 gp140-specific antibodies to bind to the complement factor C1q. A fourth correlate was of increased risk: SIVsmE543 gp140-specific antibodies that could interact with C1q. When analyzed separately, however, despite the nearly identical extents of protection, the correlate analysis yielded different results for the intramuscular (69%) and aerosol (70%) immunization groups. Thus, when the authors classified the Fc profiles of susceptible and resistant animals in the two immunization groups and further analyzed data from antibody binding and functional assays, they identified IgG-mediated phagocytosis by monocytes as a CoP in the intramuscular group but IgA-mediated phagocytosis by neutrophils as a CoP in the aerosol group. The authors then noted the contradiction between IgA as a correlate of increased risk for acquisition in the RV144 human trial but reduced risk both in the aerosol group of their macaque experiment and a passive mucosal immunization experiment (36, 51, 66).

In a macaque study of ALVAC-priming and Env protein boosting, one group received a bivalent gp120 protein boost to mimic the RV144 design, while another group was given a pentavalent gp120 boost. All of the animals were challenged with a tier-2 (NAb-resistant) simian-human immunodeficiency virus (SHIV), which was mutated in *env* to enhance the exposure of V2 epitopes (13). The infection rate in the RV144-like, bivalent gp120 group was somewhat higher than that in control animals (i.e., slightly worse than no protection), whereas there was a nonsignificant trend toward protection in the pentavalent gp120 group. A Kaplan-Meier log rank test showed significantly better protection by the pentavalent than the bivalent vaccine gp120 and when the bivalent group was pooled with the controls (a questionable *post hoc* procedure). Polyclonal serum NAbs were not detected against the challenge SHIV, but a monoclonal CD4-binding site-directed NAb, active against the challenge SHIV, was isolated from one monkey in the pentavalent group. Antibody binding to gp120 and V2 peptides, CD4 competition, and phagocytosis of gp120-coated beads were significantly stronger in the pentavalent than the bivalent vaccine group. Cox proportional hazard modeling, however, identified four other CoP candidates, all related to antibody-dependent cellular cytotoxicity (ADCC). These were ADCC peak titers, Env-specific antibody binding to cells, NK-cell-mediated ADCC activity, and antibody-mediated activation of NK cells, measured as MIP-1β intracellular expression (13).

To explore the generality of their findings, Ackerman et al. (36) incorporated the bivalent and pentavalent gp120 groups from this study (13) into their follow-up analysis. They pooled those two groups, yielding a spectrum spanning the slight enhancement of infection to an insignificant trend toward protection. Like the SIVmac239 DNA/Ad5-*env* regimens, which gave strong protection (36, 43), the ALVAC/protein immunizations were intramuscular. Analyzing serum samples from the pooled weakly or nonprotected ALVAC/protein-immunized macaques, Ackerman et al. then identified IgG-dependent phagocytosis by monocytes as the leading CoP candidate (Table 2) (36). The authors argued that immunization route-specific CoPs therefore had been corroborated. We note, however, that the proposed Ab Fc-associated CoPs in the two analyses of the weakly protected or unprotected ALVAC/gp120-vaccinated animals were not identical; one was ADCC while the other was one type of phagocytosis. From a wider perspective, it is also worth underlining that the precise Ab Fc-related CoPs that Bradley et al. identified were not exactly the same as those in the RV144 trial, where the vaccine regimen was similar to that in the unprotected bivalent-boost macaque group but where the association between ADCC and protection was contingent on low Env-specific IgA reactivity (51).

Two further studies illustrate the variable outcomes in NHP experiments. When macaques had been immunized with a DNA prime before a boost with rhesus monkey rhadinovirus, both vectors expressing near-full-length SIVmac316 genomes, they were strongly protected against acquisition of SIVmac239, a clonal, highly neutralization-resistant virus (47). Promising as that feat was, a more elaborate immunization scheme with multiple sequential viral vectors failed to provide any protection against the same challenge virus (49). In the latter study, NAbs against the highly resistant, clonal challenge virus were induced in one animal, which was nevertheless infected after one challenge (49) (Table 2). Findings like these raise questions about what factors influence protection in NHP models. How sensitive are the models to stochastic influences and subtle variation in experimental conditions? Most important, though, is the lack of protection against highly stringent challenges of vaccinated NHPs, an outcome concordant with human trials (Table 1).

**FIG 3:**
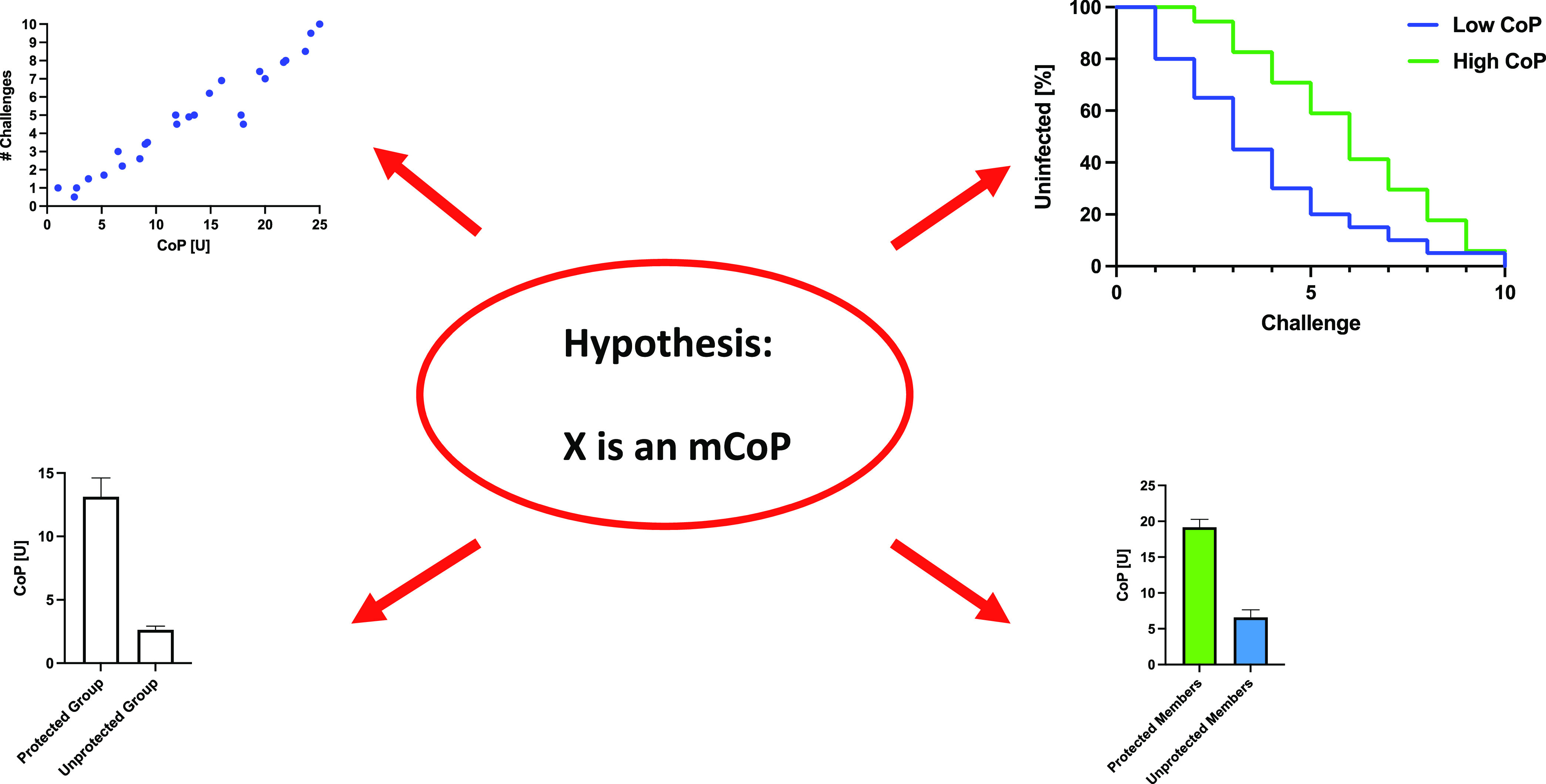
Test implications of hypothetical mCoPs. The application of systems biology in the search for CoPs and CoRs has been considered non-hypothesis-driven (98, 99). Once a factor is proposed to be an mCoP, it constitutes a hypothesis that can be tested. The arrows around the hypothesis represent implications that must hold up or the simple hypothesis is refuted. The quantified CoP (given on the *x* axis in imaginary units, U, in the upper left diagram) should correlate well with the number of virus challenges required for infection of study animals (upper right). To be relevant to protection, a CoP should be measured in samples taken close in time to the period during which protection is analyzed: causality implies stronger correlation for time-matched samples than for those from earlier or later time points. Stratification of these animals according to high CoP and low CoP values should give distinct Kaplan-Meier curves, showing more rapid infection in the low-CoP group (upper right). When there is more than one vaccine group and one shows net-protection while another does not, the measured CoP values should be higher in the protected group (lower left). Among the animals in a net-protected group or subgroup, individuals that become infected should have lower CoP values than those that stay uninfected (lower right). Even if the CoP candidate passes all those tests, however, it could still be an nCoP. Further *in vitro* experimentation is required to corroborate that the CoP is a mechanistic factor directly conferring protection. As an example, systems biological analysis of human responses to seasonal influenza vaccines showed that TLR5 expression was associated with the strength of virus-specific antibody responses. Experimentally, flagellin in murine gut microbiota was then shown to act as an adjuvant by signaling through TLR5. A similar mechanism was ultimately established in humans in that perturbing the microbiome affected the responses to influenza virus in a vaccine trial (97, 98, 169, 170).

**FIG 4:**
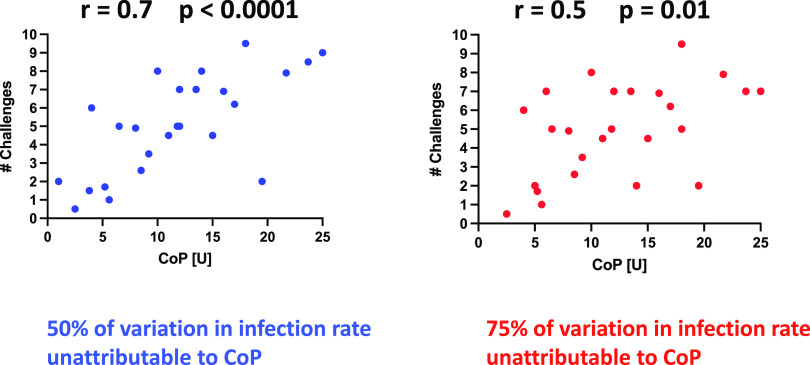
Attributable proportion of variance in parametric correlations. The simulated animal model data have passed normality tests, which legitimizes parametric correlation analyses (Pearson). An advantage of Pearson correlations is that the coefficient squared corresponds to the fraction of the variance in the number of challenges required for infection that can be attributed to the variance in the CoP values. A Pearson correlation coefficient of *r* = 0.7 (left diagram) may seem impressive, but it nevertheless leaves 50% of the variation unattributed (note that even *attributable to* is not equivalent to *caused by*). Weaker correlations, say *r* = 0.5, are often reported (note that the attribution invokes correlation strength and not its significance, which can be high for a weak correlation). The unattributed portion of the variation would then be 75% (right diagram). Often parametric correlations are illegitimate because the distribution cannot pass normality tests. Nonparametric correlations are then justified but preclude the attribution of a proportion of the variation. Thus, in spite of highly significant (*P* < 0.0001 and *P* = 0.01) and robust (*r* = 0.7 and *r* = 0.5) correlations, the extent of protection that can be attributed to the variation in a proposed mCoP may be small (50% in the left diagram and 25% in the right diagram) or even unknown when the correlations are nonparametric.

We question whether and how the various analyses of non-NAb CoPs have improved vaccine development so far. When a different CoP is identified in every other new study, it is plausible that the correlates are indirect, i.e., nCoPs (Fig. 1 and 3). A key point is that mechanistic experimentation is necessary to sift mCoPs from nCoPs. That distinction is important, because only mCoPs can directly guide vaccine design. Strong nCoPs may become valuable substitute markers for immune responses that are known through other insights to be effective (29), but even when correlations are apparently strong, only a proportion of the variation in protection can be attributed to the ostensive nCoP or mCoP (Fig. 4). Hence, a strong correlation does not exclude the possibility that another, unknown factor accounts for a greater proportion of the variation. Overall, vaccine development would benefit from the identification and comparison of all strong protective factors.

Contradictory CoP and CoR data should also serve as tests for hypotheses about mCoPs (Fig. 3). A well-quantified immune response that correlates with protection in one study but not in another, provided it reaches similar levels in the two contexts, cannot be an mCoP on its own. Examples are the non-NAbs elicited in response to gp120-protein immunizations in the VAX003, VAX004, RV144-RV152, and HVTN702 human trials and, e.g., in the macaque experiment discussed above (13) (Table 1). To explain the apparent difference in protection between VAX003/004 and RV144, the dominance of IgG2 and IgG4 responses to gp120 in the former and of IgG1 and IgG3 in the latter has been invoked. It was proposed that IgG subclass-specific effector functions could account for the somewhat different outcomes of the trials (67), but if specific IgG-subclass profiles were a central determinant of protection in RV144, analyses of the HVTN 702 trial should also show that the putatively unprotective IgG2 and IgG4 subclasses were dominant. We are not aware of any data that directly show this to be the case. Furthermore, comparisons with macaques are not straightforward, since their IgG subclasses have biophysical and functional properties distinct from those in humans (68). A model was, however, developed from RV144 data to account for personalized differences in FcγR activation (69). That model was projected onto the antibody response in HVTN 702 by incorporating expected ethnically associated differences in IgG allotype profiles. The IgG1 to -4 concentrations in RV144 were then converted to the predicted levels in individuals with the allotype distributions expected in the African trial population. The model indicated that IgG-FcR ligation would be significantly reduced in HVTN 702 compared with RV144 vaccinees because of lower IgG1 levels rather than differences in IgG3 or IgG4 concentrations (69). The sophistication of this systems biology approach does, however, rely on multilayered assumptions and unavoidable uncertainty. Any practical implications for vaccine design would be challenging to apply.

## IMMUNOLOGICAL ASSAY DESIGNS MAY INFLUENCE CoP IDENTIFICATION

ADCC is a non-NAb function that is commonly measured in CoP analyses; induction of ADCC-mediating non-NAbs is the aim of multiple past and current vaccine projects. Rightly or wrongly, ADCC is widely held to be an mCoP, but how ADCC is measured varies between studies, and that variation may contribute to inconsistencies in correlations. Controversies have arisen about which ADCC assays are relevant to natural human HIV-1 infection. Commonly used soluble forms of Env, such as monomeric gp120s or nonnative trimers, fail to present epitopes for broadly active NAbs (bNAbs) but expose narrow-NAb and non-NAb epitopes. When such Env proteins are added to cell cultures, the cells to which they bind become ADCC targets (70). As Env binds to CD4 on the cell surface, additional epitopes are exposed that are masked on functional native Env, while other epitopes are distorted. The effect is that ADCC activity can be overestimated in these *in vitro* assays compared with what occurs *in vivo*. Likewise, cells infected with HIV-1 *nef* and *vpu* mutants do not effectively downregulate cell surface CD4. Accumulated viral debris, particularly shed gp120, can bind to the remaining CD4, thereby exposing nonnative epitopes and creating spurious susceptibility to ADCC (71–74). ADCC assays that do not differentiate between virus-infected and uninfected cells, such as those based on detection of granzyme B activity in target cells or activation of the effector natural killer cells (CD107a and gamma interferon detection), can also overestimate potentially protective ADCC responses. These skews are revealed by comparisons with the specific detection of infected cells by an mRNA flow technique (74). A final point is that although ADCC-mediating antibodies often lack neutralizing activity, bNAbs generally mediate ADCC as well as non-NAbs or better, particularly in assays that simulate *in vivo* conditions (75).

Antibody-dependent phagocytosis (ADCP) is another non-NAb function (13, 36, 76–80), but it is, again, debatable which ADCP data obtained *in vitro* are relevant to protection *in vivo*. If ADCP assays register phagocytosis of inert virions or viral debris, how does that affect their relevance to protection? It has also been argued that HIV-1 virions simply evade phagocytosis because of their notoriously low spike density (76, 81–83).

In summary, there is a long-standing need to consolidate the design and interpretation of ADCC and ADCP assays aiming to identify mCoPs.

## CORRELATES OF VIREMIC REDUCTION

CoPs and other CoRs can also be sought with viral load as the endpoint. For example, in the above-described NHP study comparing intramuscular and aerosol-intranasal vaccination, where efficacy in preventing acquisition was ∼70%, peak viral loads were lower in infected animals in the intramuscular than in the intranasal immunization group (36). In principle, correlates with the spectrum of viral loads could have been sought, although in the RV144-RV152 human trial, viral loads did not differ between infected vaccine and placebo recipients (51). As yet another comparison with that clinical trial, in one study macaques received SIV *gag* DNA in combination with *env* DNA, plus either recombinant gp120(A244) and gp120(MN) as protein or *V1V2-env* DNA plus V1V2 peptides and a cyclic V2 peptide, before virus challenge; the peptides were given either intramuscularly (i.m.) or intranasally (i.n.) (37). This protocol drew on the ostensibly advantageous V1V2-focused responses in the RV144 trial. Upon challenge with the neutralization-sensitive (tier-1) SHIVBaL.P4, the widely diverging infection rates in the separate control groups precluded an evaluation of protection from acquisition. However, in contrast to the RV144 results, there was significant control of viremia in the bivalent-gp120 group (37). Antibody capture and neutralization of the challenge virus correlated with control of viral load in the group that received the gp120 proteins. Control of viremia in the V1V2 immunogen i.m. and i.n. groups, however, was not significantly stronger than that in control animals. The titers of the V1V2-reactive antibodies also did not differ between viremia controllers and noncontrollers, and Fc-dependent effector functions did not differ in this manner. The authors therefore suggested that the V2 antibodies identified in the RV144 trial are not an mCoP (in our current terminology) but instead just a surrogate marker (nCoP) for an unidentified immune response. This tentative conclusion may have some merit, although we note that mCoPs for protection from acquisition and for control of viremia need not be the same, and the weakness of any protection in the RV144 trial should preclude strong conclusions about CoP candidates.

## THE SEARCH FOR CoPs IN THE ABSENCE OF NET PROTECTION

Even when there was no overall protection from acquisition or viremic control, batteries of assays and systems-serological approaches have been applied in the hunt for CoRs and even CoPs in subgroups where there was some protection. We outline above why this kind of analysis is inherently problematic. Here, we discuss some such studies.

In the absence of protection, it is possible to identify CoRs. First, a vaccine could increase acquisition in some individuals but reduce it in others, leading to no net protection. In such a case, subdividing the vaccine recipients might allow a CoP to be identified in a subset (Fig. 1). High and low NAb titers against flaviviruses can thereby have opposing effects, but such scenarios do not apply to HIV-1 or SIV (17, 84). Furthermore, the CoR could reflect an immune or other susceptibility factor that also varies in the control group and would give even stronger correlations in both groups if it were known and quantified (29, 30). Here is an imaginary example: suppose the expression of any non-vaccine-induced infection-enhancing factor correlated inversely with completely inert, unprotective antibody responses. If so, the latter would correlate with a lack of acquisition among vaccinees, whereas the infection-enhancing factor would correlate with susceptibility among both vaccinees and controls (29).

One macaque experiment illustrates several points. A replicating adenovirus vector expressing SIV genes was used for two intranasal or intratracheal priming immunizations before two intramuscular boosts with gp120 or gp140 proteins. After iterated low-dose intrarectal SIVmac239 challenge, infection was delayed only in female macaques and mainly in females in the gp120-boosted group. No such delay occurred in male animals, and for the pool of males and females there was no protection from acquisition in any of the immunization groups (79, 85). No NAbs against the challenge virus were detected in any animals and, paradoxically, gp120-binding non-NAb titers were higher in the males than females. The influence of rectal Env-specific IgA was investigated after division of its concentration in rectal secretions into high and low categories. Kaplan-Meier acquisition curves for high and low IgA levels were then compared (>0.04 or <0.04 ng/mL), which identified high rectal Env-specific IgA as a CoP for female macaques. That correlation, however, applied to the gp140-immunized but not to the gp120-immunized female macaques, even though the latter was the best-protected subgroup. That outcome refutes the simple hypothesis of Env-specific IgA as an independent mCoP. Furthermore, if Env-specific IgA were an mCoP, its concentrations should be higher in the females than the males, but the differentially protected sexes were indistinguishable in this regard. How would the IgA then protect in one case but not the other? Rectal memory-B and plasma cells were higher in the female group than the male, but they were not higher among the more strongly protected gp120-immunized females than their gp140-immunized counterparts. It is imperative to formulate sharp hypotheses and to clarify exactly which outcomes refute or corroborate them. If entangled auxiliary hypotheses are required to shore up the initial one, they should be made explicit (Fig. 3).

In a follow-up analysis, antibody-dependent complement-mediated lysis (ADCML) of virions (but not of the arguably pivotal virus-infected cells) was found to correlate with the number of challenges required for infection of the gp140-immunized male macaques but not the gp120-immunized males or any of the females (79). The ADCML activities also were no higher among males than females (Fig. 3). Overall, the varied patterns of data for different subgroups subvert any conclusions about mCoPs that could be exploited for vaccine design.

In one of the NHP studies discussed above, anti-Gag IgG titers and levels of certain glycoforms of gp120-specific IgG were higher in females and correlated more strongly with ADCML than in males (although ADCML did not correlate with protection among females). Conversely, other gp120-specific IgG glycoforms found in males correlated more strongly with ADCML than in females. It was noted that various antibody effector functions and glycosylation profiles correlated better in males than females, while biophysical data, including Fc-receptor binding, correlated better among females (79). How these various observations would help vaccine design remains obscure. Among other uncertainties is the relevance of the *in vitro* assays for ADCML of virions only to protection *in vivo*.

It is also worth contrasting the above-described complexity with the results of another study that the same group conducted with similar immunogens, comparing sublingual, intranasal plus intratracheal, intravaginal, and intrarectal immunization routes (86). In that experiment, the vaccines tested did not protect significantly against acquisition, whether vaccine groups were pooled or compared individually with shared controls. Despite the lack of protection, the number of challenges before acquisition correlated strongly with the avidity of serum antibody binding to Env proteins and with ADCC titers of sera from the vaccinated animals. Furthermore, animals with high rectal anti-Env serum IgA (IgA) titers (>1:8) required significantly more virus challenges to become infected than those with low titers (<1:4). This apparently beneficial role of rectal sIgA, in the absence of net protection, again contrasts with the report of IgA, albeit in serum, as a correlate of increased acquisition risk of infection in the RV144 trial (51). It should also be noted that the avidity assay used in the macaque study is based on chaotrope-induced dissociation of immune complexes, which is a highly problematic method when applied to intricate, large, and metastable antigens like HIV-1 or SIV Env proteins (87, 88).

In another study, female macaques were first immunized with three different replicating Ad5hr (hr, host-range mutant) SIV vectors, each expressing *env-rev*, *gag*, or *nef*, intranasally and orally and then again intratracheally, before intramuscular gp120 boosts (SIV-CG7V and SIV-M766). After iterated intravaginal exposure to SIVmac251, the rate of virus acquisition did not differ from that in the control group by Kaplan-Meier analysis. Measures of ADCC activity, however, were found to correlate with the number of challenges required for infection of the vaccinated animals, whereas ADCP activity and FcγRIII expression in cervicovaginal macrophages did not correlate. Again, however, since there was no evidence for vaccine-mediated net protection, a counterbalancing effect must be postulated if some vaccine effects were indeed intrinsically beneficial.

Another highly complex immunization regimen consisted of first orally plus intranasally and then intratracheally administered Ad5hr SIV *env* M766 and 239, followed by intramuscular boosting with SIV M766 + CG7V gp120 proteins and ALVAC-SIVM766 or DNA vectors expressing SIV genes and rhesus interleukin-12 (IL-12) (Table 1) (89). There was no significant delay in acquisition compared with the control group after iterated SIVmac251 rectal challenges. Despite, again, the lack of vaccine-mediated protection, the proportion of Env-specific rectal IgA correlated significantly (*P* = 0.012) but very weakly (*r* = 0.35) (Fig. 4) with the number of challenges required for acquisition when male and female macaques were pooled. In males, ADNP (antibody-dependent neutrophil-mediated phagocytosis) correlated more strongly with delay of acquisition (*r* = 0.60). The changes in the gut microbiome were greater in females than in males (89). The authors concluded that these findings validated their previous report of differential, sex-dependent responses relevant to protection from acquisition (85), but the CoRs just summarized for the second study (89) were not the same as those found in the first experiment (90, 91). Thus, in the first study, the CoP for females was reported to be rectal Env-specific IgA and Env-specific memory B and plasma cells, but for males the CoR was ADCML of virions (there was no protection of the male group [85]). Do the various and conflicting data sets derived from male and female macaques really suggest that men and women would need different vaccines (79, 85, 89)? It seems imprudent, or at least premature, to draw such a consequential conclusion, and it is not obvious what the sex-specific vaccine designs should be.

Correlates of reduced risk of acquisition were sought in the HVTN 505 human trial of a DNA plus rAd5 vaccine, expressing HIV-1 genes including *env*, in the absence of protection (Table 1). Antibody-dependent monocyte phagocytosis (ADMP), antibody binding to FcγRIIa, and breadth of anti-Env IgG3 were found to correlate with reduced risk of acquisition while anti-Env IgA correlated with increased risk (80). The absence of net protection might, again, suggest that opposing factors exert effects so similar in magnitude that they cancel each other out. In other words, intrinsically protective immune responses induced by the vaccine would have to be nullified by other effects of immunization. If this is the case in this and other experiments, substantial mechanistic analyses would be needed to help vaccine redesign.

## INVOKING THE MICROBIOME IN VACCINE-MEDIATED PROTECTION

The dissection of causalities becomes even more complex when the effects of vaccination on the microbiome are invoked. In one NHP experiment, two priming immunizations with modified vaccinia Ankara (MVA) vectors expressing SIVmac239 Gag, Pol, Env, Tat, and Nef, as well as with SIV peptides and a CD4-gp120 fusion protein, followed by two oral boosts with SIV peptides and a CD4-gp120 fusion protein, led to significant protection (VE, 44%) against iterated intrarectal challenges with the neutralization-sensitive (tier-1), highly divergent SHIV-SF162.P4. No NAbs against the challenge SHIV or antibodies binding to the immunogen or challenge virus gp120 were detected in serum or the rectal mucosa, and no Env-specific B-cell responses were observed in mesenteric lymph nodes. What, then, could be responsible for the Env antibody-independent protection? A systems serology analysis identified vaccine-induced alterations and reduced bacterial species diversity in the gut microbiome. The suggested potential mCoPs were trained innate immunity, as evidenced by tumor necrosis factor alpha (TNF-α) and IL-6, as well as virus-specific T cells (92).

In principle, effects on microbiome diversity could contribute to vaccine-induced mucosal protection. Some such effects might, however, be adjuvant linked and, as suggested, yield trained innate immune responses (92). If so, they would be ancillary to the crucial adaptive responses that the reengineering of Env-based immunogens is intended to achieve; hence, it would not inform Env-immunogen design. Microbiome changes seem intrinsically too tangential to be either mCoPs or inducers of mCoPs; perhaps a more accurate description would be that they are advantageous cofactors. The core problem remains how to elicit the requisite NAb specificities. Components in the intestinal microbiota can induce antibodies cross-reactive with gp41 (89), but these antibodies are not associated with protection (93). Env proteins in general induce many specificities of non-NAbs, non-NAb epitopes even serving as immune decoys that weaken potentially protective NAb responses (61). It is not obvious why or how the induction of unprotective antibodies would be beneficial through any linkage to vaccine-induced changes in the microbiome, but in the cited study (92), virus-specific T-cell responses were the only adaptive ones possibly linked to protection.

## EXPANDED SYSTEMS BIOLOGY IN THE SEARCH FOR CoPs

Extensive systems serology searches across multiple analytes yield complex data sets, sometimes yielding valuable clues. When outcomes differ among experiments in the NHP model or between macaque and human studies, it is always prudent to ask whether a correlation is mechanistically or indirectly linked to vaccine protection or is merely a chance event.

In one systems serology analysis, when 300 variables were screened for correlations with a protective rank score in a macaque vaccine challenge model, many intricate and immunologically plausible correlates were identified (31). To aid vaccine development, however, the complex networks must be dissected to define how the innate immune, metabolic, and genetic regulatory factors are causally linked to protective adaptive immune responses or to other protective mechanisms. Sometimes methods to improve immunogenicity can be pinpointed. For example, the induction of the transcription factor CREB1 (cyclic AMP-responsive element binding protein-1) by the canarypox vector may qualitatively have improved the immune responses to Env in NHPs; it correlated with an increase in the number of SIV challenges needed for infection. Expression of CREB1 and its target genes, but also of several transcription factor targets without strong links to immunity but to more general cellular functions, correlated with antibody responses to V1V2 epitopes (94). The suggested mechanism of the CREB1 effect was cGAMP-mediated regulation, leading to recruitment of CD4^+^ T cells and B cells to sites of antigen presentation (94). The ultimate mediators of protection could therefore be adaptive responses as the last steps in branched causal chains. Hence, these approaches may yield important insights, if not for the design of Env-immunogens then into how the vector and adjuvants mold the responses. Adjuvant research is, after all, a central component of Env-protein vaccine development.

Systems biology is a powerful tool for identifying factors that foster and modulate immune responses and thereby mediate or compromise protection by vaccines (95–102). The information gleaned from such analyses of the highly complex network of intrinsic, innate, and adaptive immune functions is clearly valuable, but if mCoP candidates emerge, they must be tested experimentally and survive attempts to refute them (98, 99) (Fig. 3). However, even incontrovertible knowledge of an mCoP may not be readily translated into superior immunogen designs, such as multiple antigenic variants and multimeric presentation, or improved adjuvants.

## THE INFLUENCE OF CoP ANALYSES ON VACCINE DESIGN

The results of the RV144 study in Thailand formed the rationale for the HVTN 702 efficacy trial in South Africa about 10 years later. Although the two trials differed in many respects, they were both based on ALVAC-vector priming and monomeric-gp120 boosting (Table 1). The HVTN 702 efficacy trial explored the protective potential of a variation of the RV144 regimen as well as its capacity to elicit cellular immunity and non-NAbs. One goal was to elicit non-NAbs to the V1V2 region of gp120, since such antibodies had been identified as the major CoP in RV144 analyses (51). HVTN 702, however, failed to show any protection (3). If one accepts protection in RV144 as a fact, which of the multiple differences between the RV144 and HVTN 702 regimens could logically be responsible for the discordant outcomes?

In the HVTN 97 phase 1b immunogenicity study, the RV144 immunization regimen, including the alum adjuvant component, was exactly replicated in a South African population; the resulting V1V2 antibody responses were similar in the two trials (103). The phase 1/2 HVTN 100 trial, also in South Africa, tested the regimen that came to be adopted in HVTN 702. The ALVAC and gp120 immunogens differed from those used in RV144 and HVTN 97, and MF59 replaced alum as the adjuvant (104). In HVTN 100, the CD4^+^ T-cell responses and gp120-binding antibody titers were higher than those in RV144 and HVTN 97, but the V2-specfic IgG responses were lower (105). Either the different immunogens or the adjuvant switch could have been responsible for these distinct immunogenicity profiles. Similar immunization regimens, including the two adjuvants, were also studied in macaques. In one such ALVAC-prime, gp120-boost experiment, the VE was 44% against acquisition of the tier-2 SIVmac251 challenge virus in the vaccine-alum group but there was no significant efficacy in the vaccine-MF59 group relative to shared simultaneous and historic controls that received either adjuvant or not and a control protein or not (15). One obvious potential explanation is that the distinct efficacies are determined by the different adjuvants because of the similar contrast between RV144 (alum, partial protection) versus HVTN 702 (MF59, no protection). In other respects, however, such as differences and similarities in immune responses and the precise CoRs identified (Fig. 3), the data in these and other studies do not consistently support that interpretation.

In the above-described macaque study (15), the IgG reactivities in serum and rectal secretions measured with scaffolded V1V2 antigens (the same method as that in the CoP study of RV144 [51]), and in serum with cyclic V2 antigens, were markedly and significantly higher in the MF59 than the alum group (15). The converse was found only for rectal IgG reactivity with the cyclic V2 antigen. In contrast, in the human trials, V1V2 antibody titers were lower with MF59 (HVTN 100 and 702) than with alum (RV144 and HVTN 97), although the immunogens also differed (104, 105). It is furthermore notable that the association of borderline protection in RV144 with serum IgG V1V2-scaffold reactivity was not confirmed in the macaque study. There, the group without net protection had the stronger responses (Fig. 3). Furthermore, detectable rectal IgG binding to V2 was a CoP in the alum group but the opposite, a correlate of increased risk, in the MF59 group (15).

What may have been responsible for the different outcomes in the Alum and MF59 adjuvant vaccine groups? The authors did not invoke potential differences in precisely which V2 residues the anti-gp120 IgG antibodies interacted with or how this might matter. Instead, they hypothesized that antibody effector function variables were responsible. They reported that the Fc regions of the V2-specific IgG in the alum group were enriched for glycans that are deemed to favor ADCC and complement activation (15, 106). In contrast, the Fc regions of the V2-specific IgGs in the MF59 group showed elevated sialylation, which is linked to anti-inflammatory activity (107). Several other CoPs were also identified in the alum group, including IL-17-producing innate lymphoid cells and the expression of 12 genes, 10 of which are associated with the Ras-mitogen-activated protein kinase (MAPK) pathway (15). The latter signaling pathway links many ligand-receptor interactions to gene activations (108).

Whatever the influence of the observed differences in Fc glycosylation between the two adjuvant groups, antibodies capable of mediating ADCC, ADCP, and ADCD (antibody-dependent complement deposition) responses were consistently better induced with MF59 than with alum; the differences between the adjuvant groups were highly significant (15). The superior immunogenicity and the Fc-dependent functions associated with MF59 use may have influenced the decision to use this adjuvant in HVTN 702 more than the partial protection in the alum group. The multiple inconsistencies between the *in vitro* data and the degree of protection, within and between the macaque and human studies, must have complicated the choice.

In follow-up studies, transcriptomic analysis of RV144 samples identified IRF7 (interferon regulatory factor 7) as a CoP and the activation of mTORC1 as a CoR for increased HIV-1 acquisition (109). The induction of CREB1 also correlated with the modest protection against acquisition in RV144 (94). In contrast, CREB1 expression was reduced in the nonprotective HVTN 702 trial, where the adjuvant was MF59 instead of alum (94). The results of the macaque experiment with ALVAC-SIV plus gp120 with partial protection in the alum but not in the MF59 group also fit this pattern (15, 94). In summary, alum but not MF59 activated the Ras-MAPK pathway, which is linked to CREB1 activation (15, 94, 108). Thus, in contrast to the results of many other studies (Tables 1 and 2), the findings on CREB1 do bridge aspects of the NHP challenge model and human trials. Nonetheless, the reported differences between alum and MF59 adjuvants in whether they aid protective responses do not readily conform with some other findings. For example, MF59 induces stronger virion-capturing antibody responses than alum (110), but virion capture has been linked to both protective and infection-enhancing net effects (37, 66, 110–112) (Table 2), and alum is less effective than MF59 in supporting the induction of serum antibody responses to Env immunogens, including the anti-V2 IgG response, a reported CoP candidate (15, 16).

A later macaque experiment with the ALVAC-prime, adjuvanted gp120-boost regimen was published just before the HVTN 702 trial ended. It showed no tangible protection against vaginal challenges by the tier-1, clade C SHIV-1157ipd3N4 in the alum group. Nevertheless, IgG to V2 was reported to be a correlate of reduced infection risk in that group. In contrast, VE was 64% against acquisition in the MF59 group, where tier-1 NAb titers were a CoP (16). Despite these contrasting degrees of protection and correlations, the gp120- and V2-binding IgG reactivities in serum and vaginal secretions were indistinguishable between the two adjuvant groups. Furthermore, the serum tier-1 NAb titers, including low titers against the challenge virus, were also similar in the two groups (16). These data raise two questions. Why were equally high V2-specific responses a CoR in one group but not the other? Why did NAbs protect in one group but not the other (Fig. 3)?

Overall, the human and macaque data combined do not unambiguously identify the adjuvant switch as the explanation for the different immune responses or VEs in RV144 and HVTN 702. Other differences between the two trials could have been responsible or contributory, such as variation in immunogen, the number of boosts, the circulating viruses, and the highly diverging risks of infection in the cohorts (3). The multiyear saga of RV144, HVTN 702, and the associated NHP studies epitomizes the problems of analyzing weak to nonexistent protection against acquisition. The uncertainty of protection in RV144 must not be forgotten in comparisons of RV144 with other trials or with macaque experiments (53, 58). In the end, we may never know or understand how the different regimens in the RV144 and HVTN 702 trials affected their outcomes, but when they are weak and inconsistent, the various correlations obtained in the human and macaque studies are risky grounds for decisions on regimens for new trials.

## VARIABLE NET SUSCEPTIBILITY TO INFECTION IN THE ABSENCE OF VACCINATION

Humans and macaques vary in their susceptibility to virus acquisition in the absence of vaccination. Nonmutually exclusive, interacting variables are mucosal susceptibility, current and past other infections, the prevalence of target cells, cell surface densities of CD4 and coreceptors, and multiple aspects of intrinsic, innate, and even adaptive immunity (29, 96–99, 113, 114). In placebo groups, susceptibility may be differentially affected by the adjuvant, vector or protein controls, or the absence of these. This point is important to experimental design, since humans in the placebo groups usually are not given such control preparations; in animal experiments such controls are included more often but not systematically (43, 86, 89, 115). Indeed, in the RV144 efficacy trial, the human placebo recipients received only the virus stabilizer and freeze-drying medium (2). In HVTN 702, the placebo was 0.9% sodium chloride (3). In the VAX003 and VAX004 trials, the alum adjuvant was given to the placebo groups but not a control protein (116, 117). The omissions of control components from human trials, albeit for understandable regulatory, logistical, and cost-based reasons, might conceivably matter. When vaccine protection is weak, subtle effects on innate immunity could contribute as much to the outcome as immunogen-specific adaptive responses. Without further testing we do not know how a control (non-Env) MVA vector or adjuvant in RV144 or HVTN 702 would have molded the innate immune responses in the placebo group or otherwise influenced infection susceptibility.

In a macaque experiment discussed above, the control animals received alum, MF59, or no adjuvant, but none received a control vector or a non-Env protein (15). In this context, it is notable that the innate immune response to ALVAC infection of monocyte-derived dendritic cells engages the type I interferon-activated pathway, including IRF7 (118). Activation of that pathway, in turn, was associated with reduced risk of acquisition among RV144 vaccinees, who were the only participants receiving ALVAC (109).

We are seeing evidence emerge from COVID-19 research of how genetically and environmentally determined differences in innate immunity (e.g., interferon responses) in the upper respiratory tract influence whether SARS-CoV-2 infection becomes established (119–123). Furthermore, environmental exposure to various respiratory pathogens may confer trained immunity, i.e., innate immune memory, that affects susceptibility to SARS-CoV-2 (124–126).

Likewise, many variables may affect the acquisition of HIV-1 infection via the vaginal, penile, or rectal routes. The number of virus exposures needed to establish infection in the macaque models varies among individual animals, and some control animals remain uninfected at the end of the experiment (Table 2 and Fig. 2). Experimental animals are allocated so that the vaccine and control groups have as similar relative frequencies of known susceptibility and resistance factors as possible. Nevertheless, some variables affecting net susceptibility may not be controlled for; they may not even be known.

We reemphasize that identified adaptive CoRs (even CoPs if there is net protection) may correlate inversely with inherent susceptibility to infection, or they may correlate directly with innate protection, whether vaccine induced or not. If a correlate is vaccine induced it would of course be detected in the vaccinated animals but not in the control group, and vaccination-induced immune responses could then be statistically associated with, but not direct causes of, protection. By implication, any direct causal factors that can be identified and measured in both the vaccine and control groups would give stronger correlations than the indirect factors.

## COMPARING CoPs IN HIV-1 AND SARS-COV-2 VACCINATION

In contrast to the difficulties in ascertaining and interpreting CoPs for HIV-1 vaccines, NAbs have been consistently identified as the principal mCoP against SARS-CoV-2 acquisition or severe COVID-19 in analyses of multiple human efficacy trials and NHP experiments (32, 127–132). Antibody titers against the viral S-protein, which are quantifiable by enzyme-linked immunosorbent assay (ELISA) and related immunoassays, are generally correlated with the measurements of neutralization and are therefore nCoPs. In some studies, additional immune factors have been associated with the early and partial protection against disease conferred by a single vaccine dose (130, 133, 134), but these are negligible after the second dose (128, 135, 136). The successful evaluation of neutralizing MAbs in human trials, NHP experiments, and small-animal models reinforces that virus neutralization is sufficient for protection against COVID-19 (63, 129, 130, 133, 134, 137, 138). When virus challenges of NHPs are delayed for a long period (up to 1 year) after vaccination, however, the initially high and protective serum NAb titers had decayed markedly (∼7-fold against a D614G mutant from week 6 to week 48 [139]). Upon challenge, the animals became infected, as judged by virus replication in the upper respiratory tract, but did not develop pulmonary symptoms. The explanation may be that anamnestic immune responses, activated over a few days, curbed virus replication (139). Similar mechanisms may be in play in humans after their initial NAb responses have waned but when immune memory has been established. The use of booster immunizations to reactivate memory B cells with affinity-matured B-cell receptors should lead to the differentiation of some of them into plasma cells and the rapid rises in NAb concentrations.

The key contribution of NAbs in the initial months postvaccination is so clear that the emphasis is now not on identifying CoPs but on quantifying what NAb titers are sufficient to confer solid protection against infection or disease (129). The correlation between anti-S-protein and NAb titers is important in this context, since ELISA and other binding assays are logistically more straightforward to perform on a large scale than are neutralization assays. The goal is to define minimal binding titers that a new vaccine candidate must induce to be worth pursuing for licensure. Since formal efficacy trials with placebo groups are becoming increasingly impractical or unethical, mCoPs and nCoPs will become more valuable as predictors (128, 135, 140, 141).

The principal explanation for why CoP investigations have been far more successful, and much less controversial, in the SARS-CoV-2 than the HIV-1 field is the strong and consistent protection conferred against the former virus. The much higher efficacy of the COVID-19 vaccines eliminates the risk of analyzing what may be only noise. The SARS-CoV-2 vaccines also effectively induce NAbs active against most relevant field isolates, whereas HIV-1 vaccine candidates do not. As a result, an unambiguous principal CoP stands out, NAb titers. Systems biological analyses in general may yield clearer results when protection is robust (95). Although these methods are less needed when relevant strong NAb responses are induced, they have been applied to address whether non-NAbs might protect against neutralization-resistant SARS-CoV-2 variants. We note that a non-NAb on its own failed to protect against SARS-CoV-2 in a murine infection model (142). Vaccine failure was again found to correlate with viral resistance to NAbs (143). It remains highly questionable whether non-NAb effector functions are sufficient for protection when virus neutralization is absent.

When systems vaccinology is applied to different kinds of vaccines against other viruses with distinct antibody interactions and entry mechanisms, the approach has yielded valuable information about how these vaccines work and can be improved (96–100). Yellow fever virus, like other flaviviruses, is subject to prominent antibody-dependent enhancement of infection. NAbs can enhance infection when their occupancy on virions is too low to neutralize (17, 84). The yellow fever vaccine is a live attenuated virus, which therefore activates the immune system in a complex manner, which can be analyzed by systems biological approaches (100). Influenza virus is atypical in that antibodies to the viral neuraminidase, which do not neutralize infectious virions, still curb infection by blocking virion release from infected cells. Hence, non-NAbs may contribute more to protection against (31) influenza virus than against other viruses, and the general propensity to induce antibody responses by inactivated virions and attenuated virus can be compared and predicted by systems biological analyses (17, 97).

These various studies suggest important distinctions about systems vaccinology. The method can be applied to the identification of factors or signatures associated with reduced propensity for acquisition; thus, it may reveal multiple CoP candidates for further mechanistic validation or it may be used to dissect the complex immune system interplay that yields protective responses, e.g., NAbs (96, 98, 99, 113, 144). Correlates of the elicitation of bNAbs in HIV-1 infection, both host and viral factors, have fruitfully been identified by systems biological approaches (145–148). In the case of a highly protective SARS-CoV-2 mRNA vaccine (BNT-162b2), systems biological approaches have begun to dissect innate responses and how they pave the way for adaptive ones that ultimately elicit high NAb titers, the strong and crucial mCoP (95).

Compared with the success of the SARS-CoV-2 vaccines, even autologous protection against HIV-1 in the macaque model is modest. Immunization of macaques with stabilized HIV-1 Env SOSIP trimers alone or combined with Gag expressed from three different viral vectors resulted in substantial protection against autologous challenge, 53% without Gag and 67% with. NAb titers of >300 were deemed generally protective in the Env protein only group; the vector immunization seems to have contributed to protection by inducing cytotoxic CD8^+^ T cells (149). In an earlier study, immunization with SOSIP trimers induced a range of NAb titers against the autologous tier-2 SHIV challenge virus, leading to a clear CoP and 90% protective NAb titers of ∼500 against a pseudovirus and 30 against a live virus (150) (Table 2). The 50% protective NAb titers were ∼100 in that study and in meta-analyses of challenge after various passive and active immunizations of macaques (150–152). Those titers are very roughly 3-fold higher than the postvaccination titers that confer 50% protection from SARS-CoV-2 acquisition, although such titer values do vary depending on what neutralization assay is used (129). While there are quantitative uncertainties to resolve, NAbs against SARS-CoV-2 seem not just easier to induce than against HIV-1 but may also protect at lower titers.

## ANTIVIRAL VACCINE DEVELOPMENT SHOULD EMPHASIZE NAb INDUCTION

Here, we have focused on protective humoral responses while recognizing that some vaccines intended to induce CD8^+^ T-cell immunity are generating highly promising results (25). Neutralization is a specific well-defined antiviral activity of antibodies that is measured against virus infection in cell culture (17). NAbs, whether induced by infection or vaccination, are central to protection against infection by many viruses (18, 153). The CoPs in vaccination against SARS-CoV-2 infection or COVID-19 reinforce the importance of NAbs (32). In the HIV-1 vaccine field, passive immunization experiments in the NHP model have consistently shown that NAbs active against the SIV/SHIV challenge virus protect against it, while non-NAbs mostly fail to do so (31, 112, 150, 154–158). Effector functions mediated by the Fc region of the IgG molecule are not necessary for NAb-mediated protection of macaques from infection (159, 160). In some experiments, Fc-effector functions have modestly reduced acquisition or viremia in infected animals, but such functions of Env-binding antibodies are neither necessary nor generally sufficient for protection against acquisition (161, 162). Passive immunization with a single early-generation bNAb, VRC01, however, was ineffective overall in the antibody-mediated protection (AMP) human trial, although it was significantly protective against VRC01-sensitive isolates (163). Key quantitative and qualitative aspects of NAb-mediated protection under *in vivo* conditions need to be better understood (164).

We believe that virus neutralization is the key protective mechanism that should now be the focus of vaccination programs. It must be acknowledged that the NAb resistance of HIV-1 and its sequence variability constitute formidable problems to which there are no simple solutions. Even so, basic immunological, glycobiological, protein structural, antibody-binding, and neutralization studies have gone a long way toward identifying the obstacles. They include nonnative or unstable Env immunogens, entropic masking, glycan shielding, poor reactivity with germ line antibodies, variable-loop decoys, intrinsically poor immunogenicity, and the requirements for extensive somatic hypermutation to confer reactive breadth to HIV-1-specific bNAbs (6–12, 61). In addition, bNAb responses during HIV-1 infection appear to have a more limited role in curbing viral loads than in some other viral infections (e.g., SARS-CoV-2); bNAbs may not even protect effectively against HIV-1 superinfection (21–23, 165, 166). The strategies to overcome these obstacles are complex, but, however difficult, the quest for effective bNAb responses must continue. It is now clear that inducing only weak CD8^+^ T-cell immunity and non-NAbs in combination, or passive immunization with a single NAb (VRC01), is not sufficient to protect humans against HIV-1 infection. Recently, a long and complex series of immunizations with mRNA-delivered HIV-1–SIV virus-like particles (VLPs) and adjuvanted Env SOSIP trimers conferred substantial protection (VE = 79%) to macaques against a heterologous, neutralization-resistant SHIV (167). The main CoP was bNAbs directed to the CD4bs. It is likely that these bNAb responses were induced because the 276-glycan that shields the CD4bs was knocked out in the priming Env. ADCC gave but a borderline correlation with protection (167). That experiment further emphasizes the importance of NAbs for protection and suggests that mRNA delivery of Env proteins aids bNAb induction. Combining multispecific bNAb elicitation with the broad and effector-differentiated CD8^+^ T-cell responses triggered and maintained by cytomegalovirus-based vectors may be a particularly fruitful strategy (25).

## CONCLUSIONS

The multiplicity of putative CoPs outlined in Tables 1 and 2 suggests that surrogate or indirect correlates (i.e., nCoPs) of protection against virus acquisition are frequently identified. Directly exploiting that information for vaccine development seems impracticable. Our overriding conclusion is that identifying mCoPs, as opposed to statistical screening for CoP candidates, requires adherence to strict criteria for corroboration and then further experimental validation (98, 113). If one factor is proposed to be sufficient for protection that is observed in only one subgroup of humans or experimental animals, it must not be present at similar levels in another subgroup (Fig. 3). If CoPs are sought in the absence of net protection, a vaccine-induced factor promoting infection must be hypothesized as a counterbalance. That factor and the putative CoP should not be present at indistinguishable levels in two groups with different acquisition outcomes, and although the sex of animals can quite plausibly affect susceptibility, it may not be practicable to use complex and subtle sex-dependent differences in immune responses in the design of effective immunogens. A more feasible approach might be the pursuit of sex-neutral broadly active NAbs.

In conclusion, we see little to be gained by supporting additional studies aimed at teasing out CoPs from NHP experiments or human trials where there was weak or no overall protection, particularly where no counteracting CoRs induced by the vaccine can be identified. Such work risks identifying CoRs and nCoPs, or even mere noise, while missing mCoPs, and the nCoPs could be markers for non-vaccine-induced susceptibility factors. The track record of these analyses is poor, in that nothing has emerged as a strong and consistent CoP on which to base vaccine design. Given how hard it has been to create an effective vaccine, can multiple different immune parameters confer protection? Is it plausible that they all directly mediate protective immunity? How much is the problem exacerbated by spurious mass significance effects in individual studies or at the meta level of intense screening for large numbers of different CoP candidates? Does it matter when the placebo groups in human and NHP experiments do not receive control components of complex vaccines (i.e., vectors, adjuvants, and nonviral proteins) that are biologically active?

We have noted that protection from acquisition has sometimes been substantial in NHP experiments, whereas the same vaccine design has subsequently failed in clinical trials. If NHP models with various stringencies fail consistently as strict gatekeepers for expensive clinical trials, some substantive questions are raised. Are immune responses that protect the animals not induced in humans, or are they ineffective in humans? Are the animal infectivity models with iterated low dose not stringent enough and, hence, not a qualitatively or quantitatively realistic mimic of transmission among humans? Vaccine efficacy per low-dose challenge may be a sensitive means of detecting protection, but one high-dose challenge or a large number of cumulative challenges may better simulate human transmission quantitatively, at least for the highest risk groups. Specific models may need to reflect the different routes of entry in transmission to humans.

After so many vaccine failures throughout the past decades, serious thought must be applied to the future strategic direction of the HIV-1 vaccine field and the NHP models that have supported it for decades. We must give reported CoPs greater scrutiny, as to do otherwise might be considered a copout. When a CoP emerges, how should it best be policed? We need to tell whether we are playing the good CoP or the bad CoP when redesigning vaccines.
